# The Interplay between KSHV Infection and DNA-Sensing Pathways

**DOI:** 10.3390/v16050749

**Published:** 2024-05-08

**Authors:** Chunyan Han, Chenwu Gui, Shuhong Dong, Ke Lan

**Affiliations:** 1 State Key Laboratory of Virology, College of Life Sciences, Wuhan University, Wuhan 430062, China; 2 Department of Infectious Diseases, Frontier Science Center for Immunology and Metabolism, Medical Research Institute, Zhongnan Hospital of Wuhan University, Wuhan University, Wuhan 430071, China; 3 Taikang Center for Life and Medical Sciences, Wuhan University, Wuhan 430072, China

**Keywords:** KSHV, DNA-sensing pathways, antagonism, type I IFNs, proinflammatory cytokines, chemokines

## Abstract

During viral infection, the innate immune system utilizes a variety of specific intracellular sensors to detect virus-derived nucleic acids and activate a series of cellular signaling cascades that produce type I IFNs and proinflammatory cytokines and chemokines. Kaposi’s sarcoma-associated herpesvirus (KSHV) is an oncogenic double-stranded DNA virus that has been associated with a variety of human malignancies, including Kaposi’s sarcoma, primary effusion lymphoma, and multicentric Castleman disease. Infection with KSHV activates various DNA sensors, including cGAS, STING, IFI16, and DExD/H-box helicases. Activation of these DNA sensors induces the innate immune response to antagonize the virus. To counteract this, KSHV has developed countless strategies to evade or inhibit DNA sensing and facilitate its own infection. This review summarizes the major DNA-triggered sensing signaling pathways and details the current knowledge of DNA-sensing mechanisms involved in KSHV infection, as well as how KSHV evades antiviral signaling pathways to successfully establish latent infection and undergo lytic reactivation.

## 1. Introduction

The innate immune system is the most ancient form of host defense; it responds almost immediately to microbes (pathogen-associated molecular patterns, PAMPs) and injured host molecules (damage-associated molecular patterns, DAMPs) and invokes innate immune responses. Once a pathogen (such as a virus, bacterium, fungus, or parasite) invades a cell, a set of host pattern-recognition receptors (PRRs) recognizes pathogen-specific molecules, such as lipids, lipoproteins, sugars, and nucleic acids, and activates an innate antiviral response to eliminate the pathogen [1]. Over the past few years, several intracellular DNA sensor candidates have been identified, as well as an essential adaptor protein, STING, through which most of these sensors operate. The sensors that function in a STING-dependent manner include DAI, DDX41, cGAS, IFI16, and DNA-PK [2,3,4,5,6]. However, some sensors recognize cytosolic DNA in a STING-independent manner, such as the helicases DHX9 and DHX36, which detect DNA in plasmacytoid dendritic cells (pDCs) and trigger immune immunity via the adaptor protein MyD88 [7]. In addition, the sensing of endosomal DNA by TLR9 also occurs through the adaptor MyD88 [8]. Although these DNA sensors function through different adaptor proteins, they can activate the innate immune response by inducing the production of interferon or inflammatory factors.

Kaposi’s sarcoma-associated herpes virus (KSHV) is a large double-stranded DNA virus, belonging to the gamma herpesvirus subfamily, that has been implicated in the etiology of Kaposi’s sarcoma (KS), primary effusion lymphoma (PEL), and multicentric Castleman disease (MCD) [9,10,11]. Like other herpesviruses, KSHV has two distinct life cycle phases: latency and lytic replication. After infection, KSHV becomes latent in B lymphocytes and endothelial cells, and the viral genome is circularized into an episome and maintained in the infected nucleus [12]. The entry of foreign DNA into the nucleus of eukaryotic cells triggers numerous innate immune responses, such as the induction of type I IFNs, inflammatory cytokines, and DNA damage responses. KSHV genomic DNA can be detected in the nucleus [13,14] or in the cytoplasm due to the premature release of its genomic DNA into the cytoplasm [15]. Despite these innate immune responses, KSHV establishes lifelong persistence, suggesting that it has evolved to evade antiviral immunity. KSHV utilizes a variety of viral gene products expressed in latent or lytic replication to exert immune-evasive functions. In this review, we focus on recent developments in the molecular mechanisms underlying viral DNA sensing and the signaling pathways of the innate immune system. In addition, we discuss the role of the DNA-sensing pathway in KSHV infection and summarize recent advances in identifying the viral proteins that KSHV uses to evade innate immune responses.

## 2. Viral DNA-Sensing Pathways

DNA sensors detect viral DNA and trigger the innate immune response through distinct signaling pathways. The signals of exogenous DNA converge to adaptor proteins and are subsequently transduced to transcription factors that initiate the transcription of IFNs, cytokines, and proinflammatory cytokines. Upon detection, viral DNA primarily activates three critical signaling pathways—STING–TBK1–IRF3–interferon signaling, the NF-κB pathway, and the inflammasome pathway.

### 2.1. STING–TBK1–IRF3–Interferon Axis

The induction of the type I IFN response, which is regulated primarily at the transcriptional level by IFN regulatory factors (IRFs), is an important hallmark of the host response to pathogen invasion. Type I IFNs are mainly produced through signaling pathways that include three crucial proteins, known as STING, TBK1, and IRF3. STING is a crucial endoplasmic reticulum (ER)-located adaptor and is critical for regulating the production of type I IFNs in response to cytoplasmic DNA [16]. TANK binding kinase 1 (TBK1) is another crucial protein that is an important member of the IκB protein kinase (IKK) family that plays a key role in inducing the activation of IRF3 and IRF7, which further leads to the induction of interferon-stimulated genes (ISGs) and the production of type I IFNs. Upon receipt of a signal from DNA-sensing receptors, STING translocates from the ER to the Golgi complex and activates TBK1 [17]. Activated TBK1 promotes the phosphorylation of interferon (IFN) regulatory factor 3 (IRF3) and triggers the dimerization and nuclear translocation of IRF3, which ultimately promotes the production of type I IFNs and inflammatory cytokines [18,19].

### 2.2. Nuclear Factor-Kappa B (NF-κB) Pathway

NF-κB is a family of highly conserved transcription factors composed of several DNA-binding proteins of the Rel family, such as RelA (p65), Rel B, and c-Rel [20]. NF-κB can be found in most cell types and is involved in a wide range of biological processes, such as inflammation, immunity, differentiation, cell growth, tumorigenesis, and apoptosis. In unstimulated cells, NF-κB is sequestered in the cytoplasm and is tightly controlled by a family of inhibitory proteins known as IκBs. IκB proteins interact with NF-κB and mask its nuclear localization signal, thereby preventing its activity. In the case of microbial invasion, an inhibitor of the IKK complex is activated, resulting in the phosphorylation of the regulatory protein IκB. The phosphorylation of IκBα is a signal for its ubiquitylation and subsequent proteasome-dependent degradation by the 26S proteasome, which allows NF-κB to translocate to the nucleus and initiate the transcription of genes encoding various proinflammatory cytokines, chemokines, and cell adhesion molecules [21].

### 2.3. Inflammasome Signaling

Inflammasomes comprise another key signaling platform that responds to microbial DNA in the cytoplasm and induces the activation of inflammatory caspases and the production of the proinflammatory cytokines interleukin-1β (IL-1β) and interleukin-18 (IL-18) [22]. The transcription of pro-IL-1β and pro-IL-18 is initially regulated by NF-κB, whereas their maturation is mediated by a multimeric protein complex known as an inflammasome. Inflammasome complexes are formed after the activation of some sensor molecules containing pyrin domains (PYDs) that interact with the adaptor protein apoptosis-associated speck-like protein (ASC) through PYD–PYD association. Then, the CARD of ASC recruit procaspase-1 to the inflammasome complex. Once procaspase-1 is recruited into the inflammasome complex, it results in self-cleavage of procaspase-1 to form active caspase-1. Caspase-1 is an aspartate-specific cysteine protease that cleaves its substrates pro-IL-1β/pro-IL-18 into their mature forms, IL-1β/IL-18, and induces their release via a nonclassical secretion pathway [23].

## 3. Intracellular Sensors of Viral DNA

Intracellular sensors of viral DNA can generally be divided into three groups on the basis of their subcellular localization and expression pattern: toll-like receptor 9 (TLR9), cytosolic DNA sensors, and nuclear DNA sensors.

### 3.1. TLR9

**Figure 1:**
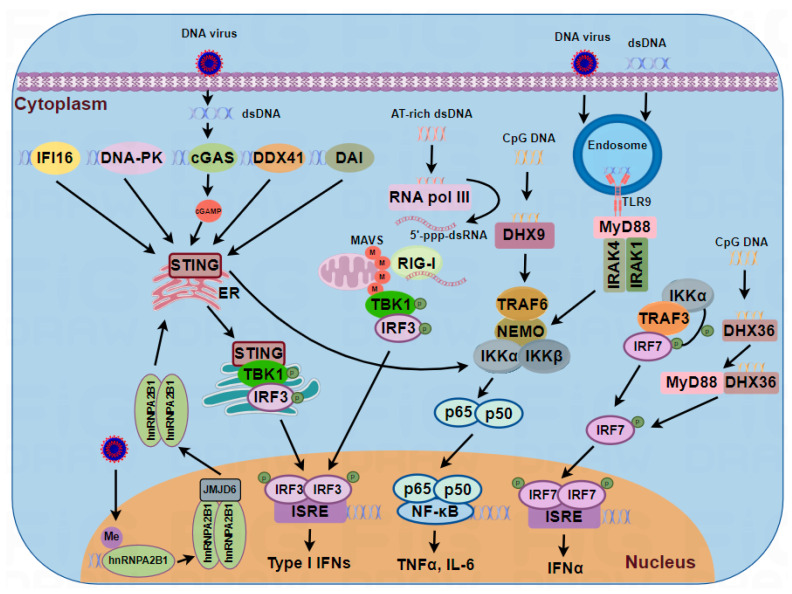
Intracellular DNA sensors involved in the activation of IFN signaling and the NF-κB pathway. The cGAS/STING pathway is a major cytosolic dsDNA-sensing pathway. Upon viral infection, cGAS dimerizes and binds to two dsDNA strands, which induces a conformational change allowing the synthesis of cGAMP from GTP and ATP. cGAMP binds to STING and recruits TBK1 to activate IRF3 for type I IFN production. In addition to cGAS, several DNA sensors, including the receptors IFI16, DNA-PK, DDX41, DAI, and hnRNPA2B1, also induce type I IFN production through the STING–TBK1–IRF3 axis. However, RNA Pol-III regulates type I IFN induction through the RIG-1/MAVS signaling pathway. Once bound to AT-rich dsDNA, RNA Pol-III will convert this DNA to 5′-triphosphate dsRNA (5′-ppp-dsRNA), which is recognized by RIG-I and interacts with MAVS to mediate type I IFN production. Aside from their role in activating IRF3, a few sensors are also involved in activating IRF7. DHX36 binds to CpG-DNA via its DEAH domain and leads to the activation of IRF7 and the release of IFN-α. In addition to IFN signaling, the cytosolic sensors cGAS and DHX9 and the endosomal receptor TLR9 can activate NF-κB signaling. After viral invasion and DNA recognition, TLR9 recruits MyD88, which induces the formation of the IRAK4/IRAK1/TRAF3/IKKα complex. This complex then triggers the activation of the NF-κB pathway and IRF7, which in turn promotes the release of proinflammatory cytokines and IFN-α. DHX9 binds to CpG-DNA and activates the IRF7 pathway, promoting the release of IFN-α.

TLR9 is a member of the toll-like receptor (TLR) family that is located on the endosomal membrane and functions mainly in immune cells, such as dendritic cells (DCs), macrophages, and B cells. TLR9 preferentially senses single-stranded DNA (ssDNA) from bacteria or viruses, particularly unmethylated CpG DNA motifs [24]. In addition to ssDNA, TLR9 has been reported to bind to DNA containing cytosine at the second position from the 5’ end (5’-xCx DNA) [25] and sense the herpesvirus genome. The interaction between TLR9 and DNA triggers one of two different signaling pathways, depending on the cell. In nonplasmacytoid dendritic cells (nonpDCs), such as conventional dendritic cells (cDCs) and macrophages, this association leads to the activation of NF-κB and AP-1, which are required for the transcription of proinflammatory cytokines, including IL-6, IL-12, and TNF [26]. However, in pDCs, the activation of TLR9 predominantly induces the production of type I IFNs [27] (Figure 1).

### 3.2. Cytosolic DNA Sensors

Cytosolic DNA sensors detect DNA in the cytoplasm of almost all cell types. Putative cytosolic DNA sensors include DAI, RNA polymerase III, DHX9/DHX36, DDX41, IFI16, AIM2, DNA-PK, and cGAS. Different sensors perform different functions. The details are shown as follows:

#### 3.2.1. DNA-Dependent Activator of IFN-Regulatory Factors (DAI)

Since DAI was proposed as the first TLR9-independent sensor for cytosolic DNA in 2007, DAI (also known as Z-DNA binding protein/ZBP-1 or DLM-1) has been extensively studied to reveal its role in antiviral immunity [2]. DAI was shown to directly bind to DNA through its Z-α and Z-β domains at the N-terminus, which preferentially sense Z-form DNA, and the D3 domain following Z-β contributes to the binding of B-form DNA. Upon recognizing DNA, DAI triggers the innate immune response through the TBK1-IRF3 and NF-κB signaling pathways. However, DAI expression has limited effects on poly(dA:dT)-induced IFN-β in mouse embryonic fibroblasts (MEFs), indicating that the function of DAI in DNA-driven innate immune responses may be dependent on cell type [28]. Moreover, in contrast to TBK1- and STING-deficient mice, mice lacking DAI displayed normal immune responses to DNA virus infection and DNA vaccines [29] (Figure 1). Thus, DAI may not function as an indispensable sensor for cytosolic DNA. To date, the role of DAI in innate immunity has not been fully explained, and the details of its mechanism need to be revealed.

#### 3.2.2. RNA Polymerase III

RNA pol III, known as a eukaryotic RNA polymerase, is considered to be an intracellular DNA sensor that functions as a stimulatory ligand for RIG-I by transcribing AT-rich dsDNA into a RIG-I-stimulating AU-rich RNA intermediate and inducing a type I IFN response [30,31]. Due to the requirement for AT-rich sequences, RNA pol III is the only intracellular DNA sensor that recognizes DNA in a clearly sequence-dependent manner. At least 30 bp poly(dA:dT) must be detected by RNA pol III to induce an IFN response in a manner dependent on the RIG-I-MAVS pathway [31] (Figure 1).

#### 3.2.3. Interferon-Inducible Protein 16 (IFI16)

**Figure 2:**
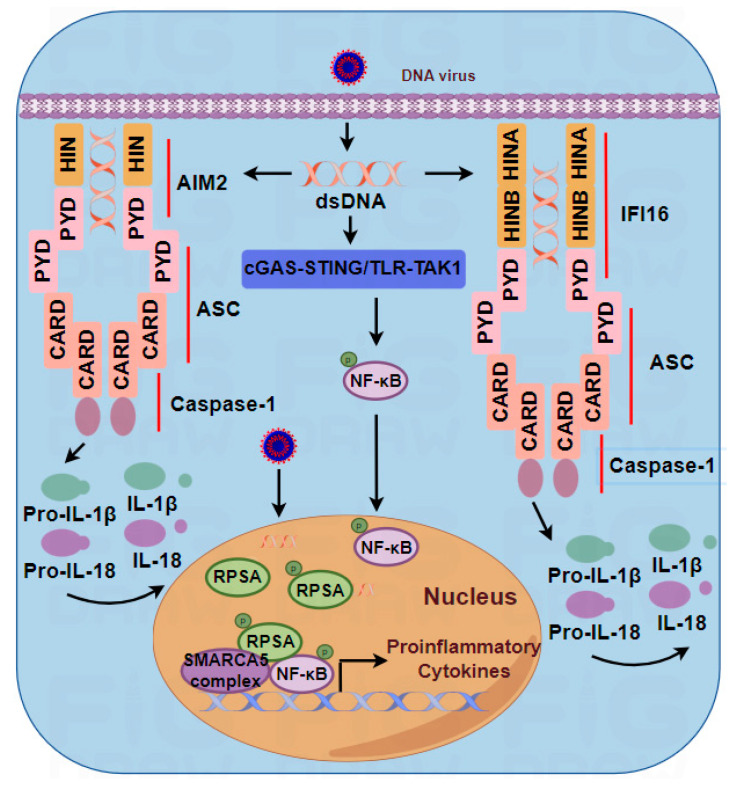
The activation of proinflammatory cytokines. Upon DNA virus infection, AIM2 and IFI16 recognize dsDNA via their HIN-200 domains. Once bound to DNA, they recruit the adaptor protein ASC through their PYD domains, which recruits pro-caspase-1 via its CRAD domain, resulting in the formation of the inflammasome complex. Inflammasome complex formation initiates the activation of caspase-1. Then, caspase-1 cleaves pro-IL-1β and pro-IL-18 into their mature forms, IL-1β and IL-18, respectively. In addition to AIM2- and IFI16-induced inflammasome activation, proinflammatory cytokines can also be upregulated by nuclear RPSA. Upon DNA virus infection, nuclear RPSA is phosphorylated. Phosphorylated RPSA interacts with the SMARCA5 complex and synergistically promotes proinflammatory cytokine transcription via the activated transcription factor NF-κB.

IFI16, a member of the human interferon (IFN)-inducible p200-protein (PYHIN) family of proteins, was initially reported as a human IFN-γ-inducible gene and a DNA damage response protein in the nucleus [32,33]. Recently, it was identified as a DNA sensor using vaccinia virus (VACV) DNA pulldown from cytoplasmic extracts of human monocytes and shown to initiate the innate immune response [3]. Upon DNA stimulation, IFI16 directly binds to DNA and recruits STING to activate the TBK1–IRF3 axis, which in turn triggers type I IFNs and cytokine production [3,34]. In addition to its role in regulating the type I IFN signaling pathway, IFI16 also activates the inflammasome pathway in the nucleus. It has been suggested that immune cells and nonimmune cells trigger different IFI16 DNA-sensing patterns. These differences are likely attributed to intrinsic cell type-dependent processes. Immune cells, such as macrophages and DCs, play a vital role in the onset of defense against viral infection. During pathogen invasion, IFI16 may localize to the cytoplasm to rapidly sense foreign nucleic acids (Figure 2). In contrast, nonimmune cells have various functions and may maintain IFI16 in the nucleus to regulate and control viral infection events.

#### 3.2.4. Absent in Melanoma 2 (AIM2)

AIM2 is a cytosolic innate immune receptor that responds to dsDNA from both the host and pathogens to trigger inflammasome activation [35]. AIM2 binds to DNA through its HIN-200 domain. Beyond dsDNA, both ssRNA and RNA:DNA hybrids engage with the HIN terminus. This facilitates the migration of AIM2 and ASC into the cytoplasm, setting the stage for inflammasome assembly. The activation of caspase-1, in turn, triggers the maturation of the proinflammatory cytokines IL-1β and IL-18 [36,37,38] (Figure 2). Although AIM2 recognizes DNA-RNA hybrids, not all DNA-RNA hybrids bind to AIM2. Therefore, it is important to understand which sequences, structures, or characteristics of DNA–RNA hybrids activate the inflammasome.

#### 3.2.5. Cyclic GMP-AMP Synthase (cGAS)

Most cytosolic DNA sensors in mammals transmit signals through the adaptor protein STING to induce IFNs in response to dsDNA [39]. Using a cell-free assay, Chen and colleagues identified a cyclic dinucleotide, cGAMP, as a second messenger that can bind to and activate STING to induce type I IFNs [40]. Subsequently, they performed biochemical purification coupled with quantitative mass spectrometry to identify the signaling molecule mediating cGAMP, and finally, they identified cGAS as a cytosolic DNA sensor that synthesizes cGAMP in a DNA-dependent manner [6]. Structurally, cGAS contains a single domain of a nucleotidyl transferase and two dsDNA-binding domains. cGAS binds DNA through electrostatic interactions and hydrogen bonds with the sugar-phosphate backbone of both DNA strands across the minor groove, explaining the sequence independence of cGAS-dependent DNA sensing and the requirement for dsDNA [41,42,43]. Once microbial dsDNA binds to the active site of cGAS, a conformational change occurs in the C-terminus, which then catalyzes the formation of a unique isomer of cGAMP from ATP and GTP [6,44]. This isomer, cGAMP, binds to the active site of STNG and causes conformational changes. Then, STING is activated by cGAMP and transported from the ER to the Golgi complex through the ER–Golgi intermediate compartment where it assembles into punctate structures that contain the kinase TBK1. During this trafficking process, TBK1 is activated. Activated TBK1 induces the phosphorylation of IRF3 and activation of the IKK complex. In brief, phosphorylated IRF3 dimerizes and translocates into the nucleus, where IRF3 binds to IFN-encoding genes and induces the production of IFNs [19,45,46] (Figure 1). The activated IKK complex phosphorylates IκB and induces the release and translocation of NF-κB into the nucleus, after which NF-κB binds to IFN-associated genes and induces inflammatory cytokines [39].

#### 3.2.6. DExD/H-Box Helicases

The DExD/H-box helicase (DDX) protein family includes a large number of RNA helicases. Members of this family have been shown to mediate gene regulation at multiple points, including signal-transduction pathways, pre-mRNA splicing, and transcriptional regulation. Recently, several DExD/H-box helicases, such as RIG-I and MDA5, and DNA sensors, have been shown to be involved in antiviral immunity by functioning as RNA sensors [47]. It was shown that knockdown of DDX41 expression impaired the production of IFNs and proinflammatory cytokines in response to dsDNA in murine myeloid DCs (mDCs) and human monocytic THP1 cells [4]. In addition to binding to DDX41, DHX9 and DHX36 bind to CpG DNA and interact with the TIR domain of MyD88, triggering MyD88-dependent TNF-α and IFN-α responses [7]. Intriguingly, in addition to its role in DNA sensing, DHX9 can also interact with dsRNA and induce MAVS-dependent IFN and cytokine expression in myeloid DCs [48] (Figure 1). This raises the question of how the same protein can detect different types of nucleic acid ligands and then induce signaling through different pathways.

#### 3.2.7. DNA-Dependent Protein Kinase (DNA-PK)

DNA-PK, which is known to be involved in the nuclear DNA damage response, is a heterotrimeric complex composed of the catalytic subunit DNA-PKcs and a heterodimer of Ku70 and Ku80. To facilitate DNA repair, the Ku heterodimer binds directly to the free ends of DNA, leading to the recruitment of DNA-PKcs to damaged sites of DNA, promoting DNA double-strand break (DSB) repair through the nonhomologous end joining (NHEJ) pathway [49,50]. In addition to its established role in nonhomologous end joining (NHEJ), DNA-PK is also implicated in cytosolic DNA sensing and the activation of innate immunity. DNA-PK can trigger innate immunity through both STING-dependent and STING-independent pathways, and which pathway is activated depends on the cell type [51,52] (Figure 1).

### 3.3. Nuclear DNA Sensors

Previously, IFI16 was characterized as the first DNA sensor to function within the nucleus, detecting herpes viral DNA in human primary fibroblasts and triggering innate immunity in a STING/IRF3-dependent manner [53]. However, the role of IFI16 in nuclear DNA sensing is controversial. Subsequently, DNA-PK, a nuclear localized protein, emerged as a DNA sensor that activated the innate immune response through the STING–TBK1–IRF3 pathway. However, it senses DNA in the cytoplasm but not in the nucleus [5]. Recent studies revealed the presence of cGAS in the nucleus, where it interacts with centromeres and long interspersed nuclear element (LINE) DNA repeats, where it produces cGAMP to induce innate immunity in human monocyte-derived DCs [54,55]. Nevertheless, compared with exogenous cytosolic DNA, nuclear cGAS exhibits significantly reduced activity toward self-DNA. In DCs and macrophages, nuclear cGAS promotes IFN gene expression by associating with protein arginine methyltransferase 5 (PRMT5), facilitating the ability of IRF3 to promote IFN production [56].

In 2019, Cao et al. explored novel viral DNA sensors in the nucleus that recognize viral DNA and then translocate to the cytoplasm to activate the TBK1–IRF3 pathway [57]. Among these sensors, hnRNPA2B1 was identified as a potential nuclear DNA sensor, challenging the notion of nuclear immune privilege concerning DNA [58] (Figure 1). Although hnRNPA2B1 is thought to be a nuclear sensor for type I IFN induction, there are no studies indicating whether and how viral nucleic acids are recognized in the nucleus to selectively induce the transcription of proinflammatory cytokine genes to enhance the antiviral innate response. In 2023, Cao et al. discovered ribosomal protein SA (RPSA) as a nuclear innate sensor that selectively induces proinflammatory cytokine gene transcription upon the detection of nucleic acids from HSV-1 and IAV. Phosphorylated RPSA coordinates with the imitation switch (ISWI) chromatin remodeling complex to regulate chromatin accessibility and cooperatively activates the expression of inflammatory factors via the activated transcription factor NF-κB [59] (Figure 2).

DNA sensors involved in the activation of IFN signaling and the NF-κB pathway are shown in Figure 1, while Figure 2 depicts DNA sensors that activate proinflammatory cytokines.

## 4. Sensing of DNA during KSHV Infection

The innate immune system is the first line of defense against pathogen invasion, and the induction of type I IFNs or inflammatory cytokines is critical for host innate defense mechanisms. Upon infection with KSHV, diverse DNA sensors initiate DNA-sensing pathways, which ultimately induce the production of IFNs and inflammatory cytokines through DNA sensor–adaptor–transcription factor cascades. Here, we will review the DNA-sensing mechanisms involved in KSHV infection.

### 4.1. IFI16 Senses KSHV Genomic DNA

IFI16 is responsible for the nuclear sensing of KSHV genomic DNA. IFI16 colocalizes with the KSHV genome in the nucleus of infected HMVEC-d cells and B cells and induces inflammasome activation [13,14]. During early KSHV infection (2 h), IFI16 forms a complex with ASC and caspase-1 in the nucleus. However, when exposed to ultraviolet (UV)-inactivated virus, IFI16 fails to form complexes with ASC. Interestingly, in later stages of KSHV infection, most of the activated caspase-1 is translocated from the nucleus to the cytoplasm, and ASC, caspase-1, and IFI16 are also redistributed in the perinuclear area [13]. However, the reason for this subcellular redistribution in response to the KSHV genome is unclear.

Excessive activation of inflammasomes in the nucleus induces widespread negative impacts on cells and tissues, including exacerbating inflammatory responses and causing cell death and dysfunction. Therefore, subcellular reorganization of IFI16, ASC, and Caspase-1 may help to prevent excessive inflammasome activation and maintain cellular homeostasis and health.

It has been suggested that large amounts of viral DNA in the nucleus can also be sensed as aberrant extrachromosomal DNA, which activates the DNA damage response (DDR) signaling pathway. Recently, an increasing number of studies have shown that IFI16 can form complexes with BRCA1 and BRCA1-H2B. These complexes recognize the KSHV genome in the nucleus, leading to BRCA1-mediated recruitment of p300 and acetylation of IFI16 and H2B by p300. IFI16 acetylation results in the formation of the BRCA1–IFI16–ASC–procaspase-1 inflammasome in the nucleus, which subsequently translocates to the cytoplasm. After cytoplasmic translocation, acetylated IFI16–H2B–BRCA1 binds to cGAS and STING, resulting in TBK1 and IRF3 phosphorylation, p-IRF3 nuclear translocation and IFN-β production [60,61]. In addition to leading to the induction of the innate interferon and inflammasome pathways, IFI16 directly binds to KSHV gene promoters and silences KSHV gene expression through epigenetic modification. IFI16 can interact with H3K9MTases, SUV39H1, and GLP in the nucleus and recruit these MTases to the KSHV genome, resulting in H3K9 methylation and KSHV lytic gene silencing [62].

### 4.2. DExD/H-Box Helicases Sense KSHV Genomic DNA

Retinoic acid-inducible gene I (RIG-I), a member of the DExD/H box helicase family, is regarded as a cytosolic RNA helicase sensor that plays a significant role in the induction of type I IFN responses following viral infection. Previous studies have shown that RIG-I can also sense DNA viruses in an RNA Pol III-dependent manner, transcribing cytosolic AT-rich DNA from DNA viruses into RNA, which is then recognized by RIG-I and activates innate immunity [30,31]. Interestingly, a recent study revealed that KSHV can activate a canonical RNA-sensing pathway. Many RNA regions in KSHV (ORF8 10420-10496, repeat region (LIR1) 119059-119204, and ORF25 43561-43650) bind to RIG-I and stimulate RIG-I-dependent, but RNA Pol III-independent, IFN-β signaling [63]. These RNA regions bound to RIG-I have no obvious sequence similarity but are highly structured. However, the details of their structure remain to be investigated.

To determine whether other DExD/H box helicases can affect KSHV lytic reactivation, a knockdown screen revealed that DDX24 and DDX49 have antiviral activity [64]. DDX24 predominantly localizes to the nucleus and targets KSHV miRNAs [65]. In contrast, nuclear localization of DDX49 plays a crucial role in regulating RNA transcription, RNA stability, and efficient export of nonspliced poly(A)+ RNAs from the nucleus [66]. Notably, since most KSHV lytic transcripts are not spliced and require nuclear export facilitated by ORF57 [67], the presence of DDX49 suggests that a critical recognition process occurs within the nucleus. Furthermore, KSHV encodes ORF10, which inhibits host cellular spliced mRNA export from the nucleus, underscoring the intricate interplay within viral–host dynamics [68].

### 4.3. cGAS Senses KSHV Genomic DNA

KSHV infection was shown to activate the cGAS–STING pathway [69]. Considering that cGAS and STING mainly recognize cytoplasmic DNA, how can viral DNA distributed in the nucleus be recognized within the cytoplasm? Several different hypotheses have been proposed to reveal how the cGAS–STING pathway recognizes DNA distributed in the nucleus. First, during KSHV lytic replication, newly synthesized viral DNA is incorporated into the capsid in the nucleus and subsequently transferred to the cytoplasm. During this process, incomplete capsids may release viral DNA, promoting recognition by cGAS and initiating the cGAS–STING–IRF3 signaling cascade [70]. Second, during apoptosis or other types of cell death, viral or cellular DNA released to the cell membrane can activate cGAS, thus stimulating innate immune responses [15]. Third, cytoplasmic accumulation of DNA fragments from virus-induced DNA damage can activate DNA sensing mechanisms [71]. Finally, virus-induced mitochondrial DNA (mtDNA) instability also induces a cGAS–STING–IRF3-mediated antiviral innate immune response [72]. In summary, the cGAS–STING signaling pathway serves as a pivotal defense mechanism against viral infections, as well as a surveillance system for genomic instability and cellular damage. Through the detection of cytosolic DNA, the cGAS-STING pathway helps orchestrate immune responses essential for host defense, immune regulation, and maintaining cellular integrity. Dysregulation of this pathway can contribute to autoimmune diseases, inflammatory disorders, and cancer, emphasizing its significance in cellular homeostasis and immunity.

## 5. KSHV Antagonizes DNA-Sensing Pathways

**Figure 3:**
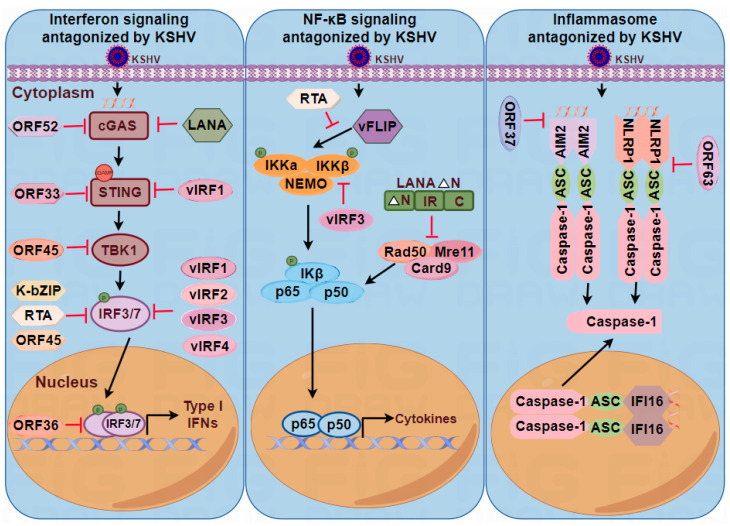
Overview of the inhibition of DNA-sensing pathways by KSHV viral proteins. Left: KSHV-encoded viral proteins antagonize the cGAS–STING–TBK1–IRF3/7-interfering signaling pathway. Middle: KSHV proteins antagonize the NF-κB signaling pathway. Right: KSHV proteins antagonize the inflammasome signaling pathway. The solid red lines indicate inhibition.

KSHV, as well as other herpesviruses, can cause lifelong infections in its host. The ability of KSHV to persist in the host relies heavily on its capacity to evade and counteract host antiviral defenses by targeting crucial cellular pathways. One such pathway is the DNA-sensing mechanism, which is vital for detecting viral DNA and initiating antiviral responses. However, KSHV has evolved sophisticated strategies to counteract this surveillance system, enabling its survival and replication within host cells (Figure 3). Understanding the mechanisms by which KSHV antagonizes DNA-sensing pathways is critical for developing effective therapeutic strategies.

### 5.1. KSHV Antagonizes the Type I IFN Signaling Pathway

#### 5.1.1. KSHV Antagonizes cGAS and STING

KSHV encodes various viral proteins that antagonize the cGAS–STING signaling pathway, including the tegument proteins ORF52 and ORF33 and the N-terminally truncated LANA isoform. KSHV ORF52 is an abundant gamma-herpesvirus-specific tegument protein that can interact with cGAS and inhibit the transcriptional activation of IFN-β mRNA [73]. Mechanistically, the KSHV inhibitor of cGAS (KicGAS), encoded by ORF52, undergoes self-oligomerization and binds to dsDNA, thereby inhibiting the association between DNA and cGAS. Upon binding to DNA, KicGAS forms liquid droplets and inhibits the DNA-induced phase separation and activation of cGAS. This finding revealed a novel mechanism by which KSHV targets host protein phase separation to suppress DNA sensing [74].

During KSHV de novo infection, the tegument protein ORF33 emerges from the KSHV virion into the cytoplasm. ORF33 directly interacts with STING and recruits PPM1G, resulting in enhanced dephosphorylation of p-STING, impaired recruitment of IRF3, and inhibition of type I IFN production. When KSHV is reactivated from latency, newly synthesized ORF33 may also promote viral lytic replication by recruiting PPM1G to dephosphorylate p-STING, thereby suppressing host antiviral activities [75].

Latency-associated nuclear antigen (LANA) is encoded by KSHV ORF73, a latent viral protein that is essential for the maintenance of KSHV latency [76]. Depletion of LANA by an inducible protein knockdown approach induced rapid degradation of viral genomic DNA. Knockdown of cGAS, STING, and other autophagy-related genes rescued the degradation of viral genomic DNA after LANA depletion. These findings revealed that LANA plays a vital role in preventing KSHV episomes from being sensed by the cGAS–STING pathway [77]. Full-length LANA is localized and functions in the nucleus of latently infected cells; however, the N-terminally truncated LANA isoform is localized in the cytoplasm and can interact with cGAS directly. Upon binding to cGAS, the truncated LANA isoform inhibited cGAS–STING-dependent phosphorylation of TBK1 and IRF3, thereby promoting the reactivation of KSHV from latency [78]. The inhibitory role of LANA cytoplasmic isoforms in DNA sensing extends the function of LANA in lytic replication and could provide an explanation for the existence of a lytic LANA promoter.

#### 5.1.2. KSHV Antagonizes IRFs and IFNs

The transcription of type I IFNs is mainly controlled by two IRFs (IRF3 and IRF7). Constitutively expressed IRF-3 is activated upon initial viral infection and induces IFN-β expression. This initial induction of IFN-β stimulates the type I IFN receptor, leading to the activation of IRF-7, which effectively induces the IFN-α and IFN-β genes, initiating a positive feedback loop [79,80].

Viral interferon regulatory factors are a group of proteins that are homologous to the cellular transcription factors of the interferon regulatory factor (IRF) family. KSHV encodes four vIRFs, vIRF1, vIRF2, vIRF3, and vIRF4. All of these vIRFs play a vital role in interfering with the interferon signaling pathway. vIRF1, encoded by KSHV ORFK9, exerts a broad inhibitory effect on IFN-β production in endothelial cells. This viral protein employs diverse strategies to inhibit IFN-β activation. First, vIRF1 interferes with IRF3 and the coactivator CBP/p300, effectively inhibiting the formation of transcriptionally active IRF-3–CBP/p300 complexes and thereby reducing IFN-β expression [81]. Second, vIRF1 directly binds to STING, preventing its interaction with TBK1 and thereby reducing STING phosphorylation and consequent IFN-β production [69]. vIRF2, which originates from KSHV ORFK11 and ORFK11.1, suppresses the transcriptional activation of IFN-β by interacting with IRF1 and IRF3. It also decreases IFN-α production through its interaction with IRF2. Mechanistically, vIRF2 disrupts the type I IFN-driven antiviral response by recruiting caspase-3 to IRF3, leading to IRF3 degradation. Furthermore, vIRF2 interfaces with p65 to inhibit the early expression of proinflammatory cytokines [82,83]. In contrast to vIRF1 and vIRF2, vIRF3 (encoded by ORFK10) regulates the production of both IRF3- and IRF7-mediated type I IFNs [84]. However, vIRF4 (encoded by ORFK10) selectively interacts with IRF7, suppressing the IFN-α-associated signaling pathway by impeding IRF7 dimerization [85]. In summary, all four vIRFs interfere with the production of IFNs.

In addition to vIRFs, the immediate early (IE) genes encoded by KSHV, including bZIP, ORF45, and RTA, are required to regulate viral lytic reactivation and antagonize innate immunity. bZIP is encoded by KSHV K8, which binds tightly to the IRF3 binding site of the IFN-β promoter and thus prevents IRF3 binding [86,87]. KSHV ORF45 is a viral tegument protein that interacts with IRF7 to inhibit both its phosphorylation and its nuclear accumulation. This interaction effectively suppresses the induction of type I IFNs [88]. IRF7 is phosphorylated and activated by IKKε and TBK1 upon viral infection. However, during KSHV infection, ORF45 is efficiently phosphorylated at Ser41 and Ser162 by IKKε and TBK1, thereby competing with the associated IRF7 and inhibiting its phosphorylation by IKKε or TBK1 [89]. In addition to ORF45, another IE gene, RTA, also blocks IRF7-mediated IFN-α and IFN-β mRNA production. RTA promotes IRF7 ubiquitination and degradation in a proteasome-dependent manner [90].

MyD88 is an adaptor for all TLRs and mediates the production of inflammatory factors and IFNs. RTA can act as an E3 ligase to degrade MyD88 via the ubiquitin-proteasome pathway and block the TLR signaling pathway. These findings provide a potential mechanism by which KSHV evades innate immunity [91].

Herpesvirus-encoded ORF36 is a conserved serine/threonine viral protein kinase (vPK) that plays an important role in regulating the viral life cycle and evading host immunity [92]. To counteract the antiviral immune response, ORF36 specifically binds to the activated form of IRF3 in the nucleus, inhibiting the interaction between IRF3 and the cotranscriptional activator CBP, which, in turn, inhibits the recruitment of RNA polymerase II to the IFN-β promoter and ultimately reduces IFN-β production [93].

RIF, the product of ORF10, blocks IFN signaling by forming inhibitory complexes that contain IFNAR subunits, the Janus kinases Jak1 and Tyk2, and the STAT2 transcription factor. This in turn inhibits Tyk2 and Jak1 activation and affects the phosphorylation of STAT2 and STAT1, which, in turn, leads to the failure of ISGF3 to accumulate in the nucleus [94]. The presence of viral genes that block IFN induction highlights the importance of the IFN pathway in the control of KSHV infection.

### 5.2. KSHV Antagonizes the NF-κB Signaling Pathway

NF-κB plays a pivotal role in the innate immune response, acting as a key regulator of various genes involved in innate immunity. During viral infection, NF-κB is activated to induce proinflammatory cytokines and chemokines. However, a number of KSHV products, such as LANA, vFLIP, and vIRF3, modulate NF-κB to evade host immune surveillance, promote cell survival, and enhance viral replication. Cytosolic LANA may antagonize cGAS, thereby blocking the interferon response triggered by cGAS. Recent studies have shown that cytoplasmic LANA isoforms also regulate the activation of the NF-κB pathway. The cytoplasmic LANA isoform recruits the MRN (Mre11-Rad50-NBS1) repair complex members Rad50 and Mre11 to the cytoplasm and inhibits Rad50–Mre11–CARD9-dependent activation of NF-κB [95]. Modulation of cGAS-dependent type I IFN responses and NF-κB activation demonstrated the importance of LANA in promoting KSHV lytic replication.

vFLIP (viral FLICE inhibitory protein) is encoded by KSHV ORF71 and is a homologous protein of cellular FLIP. vFLIP functions as a potent activator of NF-κB signaling, which is required for viral latency, survival, and tumorigenesis in PEL cells [96]. To activate the canonical NF-κB pathway, vFLIP directly binds to IKKγ and activates the IKK complex [97]. Virus-induced NF-κB activation is important for maintaining KSHV latency and inhibiting KSHV reactivation. It has been shown that KSHV RTA is necessary for initiating KSHV reactivation. This RTA-induced reactivation may be achieved by suppressing NF-κB activation. This effect is facilitated by the recruitment of the cellular ubiquitin E3 ligases RAUL and Itch by RTA, which leads to the degradation of vFLIP, thereby impeding the expression of NF-kB-responsive genes during lytic reactivation [98]. Controversially, a growing number of studies have shown that vFLIP may have a reverse impact on NF-κB, helping viruses evade innate immunity. vFLIP is able to promote A20 expression in endothelial cells and PEL cells, whereas A20 blocks NF-κB activation through the cooperative activity of its two ubiquitin-editing domains [99]. A20 negatively regulates the NF-κB pathway through disassembling K63-linked ubiquitin chains that bind to the essential adaptor RIP and mediate the degradation of RIP through the addition of the K48-linked ubiquitin chain to RIP [100]. Notably, this double-edged sword may favor viral survival and propagation in humans, where transient or low activation of NF-κB may lead to lytic replication of KSHV, whereas constitutive or persistent activation of NF-κB may lead to KSHV latency, tumor formation, and maintenance.

vIRF3, a homolog of cellular IRF, not only mediates the production of IFNs but also inhibits NF-κB activity and NF-κB-dependent transcription in a dose-dependent manner. In vivo studies revealed the ability of vIRF3 to inhibit IKKβ activity, which, in turn, reduced IKKβ phosphorylation. Furthermore, vIRF3 subtly disrupts the nuclear translocation of NF-κB, thereby affecting its antiviral function [101].

### 5.3. KSHV Antagonizes the Inflammasome Signaling Pathway

The inflammasome is a protein complex consisting of a sensor, an adapter (ASC), and an effector (caspase-1) that promotes the maturation and release of proinflammatory cytokines in response to infections. Studies have shown that three groups of inflammasomes are involved in antiviral immunity: the NLRP (nucleotide-binding domain leucine-rich repeat-containing family) inflammasome, the RIG-I inflammasome, and the AIM2 inflammasome. Among these, AIM2 primarily senses DNA viruses, RIG-I mainly senses RNA viruses, and NLRPs sense both viral DNA and RNA.

Modulation of NLR-mediated innate immunity is important for the lifelong persistence of herpesviruses. Recently, a study revealed that ORF63, a novel KSHV tegument protein, directly interacts with the NLR family members NLRP1, NLRP3, and NOD2 but shows sequence similarity only to NLRP1 and disrupts the association between NLRP1 and procapase-1, thereby inhibiting procaspase-1 processing and the secretion of IL-1β and IL-18 [102,103].

The SOX protein, encoded by KSHV ORF37, interacts with the AIM2 HIN domain through the C-terminal motif VII region and disrupts AIM2:dsDNA polymerization and ASC recruitment and oligomerization. The finding that SOX suppresses AIM2 inflammasome activation and pyroptosis, thereby promoting KSHV lytic replication, and reveals a unique mechanism for the evasion of inflammasome activation during the KSHV lytic cycle [104].

## 6. Conclusions and Perspectives

Over the past decade, we have witnessed tremendous advances in understanding the recognition of pathogen-derived nucleic acids and their role in initiating host defense responses. Diverse approaches in biochemistry and genetic and structural biology have been used to identify potential DNA sensors and reveal their unique signaling mechanisms. Each DNA sensor plays a specific role in mediating innate immunity in response to pathogen DNA invasion. However, the role and the mechanism of the DNA-sensing pathway in KSHV infection have not been fully revealed.

Nuclear IFI16 and several DExD/H-box helicases can sense KSHV genomic DNA in the nucleus. The mechanism by which IFI16 senses KSHV genomic DNA suggests that nuclear sensors may sense viral DNA in two ways. First, activated nuclear sensors shuttle to the cytoplasm to initiate the cGAS–STING signaling pathway or the inflammasome pathway. Second, these activated nuclear sensors may directly interact with chromatin epigenetic regulatory factors, mediate their epigenetic modifications, and then transport them to the cytoplasm, thereby participating in virus sensing. Although new nuclear DNA sensors have been identified, it remains unclear whether they play a role in KSHV infection.

In addition to being sensed in the nucleus and translocated to the cytoplasm, viral DNA can activate the cGAS–STING pathway directly in the cytoplasm if it is released from incomplete capsids or as a result of other virus-induced damage. However, the underlying mechanisms need to be investigated. DNA sensors normally recognize DNA, but there are also RNA sensors that recognize viral DNA. After entry into the host cell, viral DNA needs to be transcribed into RNA. These RNAs, including viral genome mRNAs, IncRNAs, microRNAs, and other viral RNAs, can be recognized by RNA sensors in the host cell. During KSHV reactivation, several members of the DExD/H-Box helicase family can sense highly structured viral RNA fragments, microRNAs, or nonspliced RNAs, but the exact mechanism involved remains to be investigated.

Although diverse DNA sensors or RNA sensors have been reported to recognize KSHV nucleic acids, further research is needed to elucidate how KSHV or other DNA viruses are controlled by innate immune sensors and cells. For example, how do different nucleic acid-sensing pathways function in the same cell, and how do such pathways crosstalk? How does DNA sensing distinguish between self and non-self DNA in the nucleus? What is the relationship between the nuclear sensing of DNA and the sensing of DNA damage? How are RNA sensors activated during dsDNA virus infection, and how do the DNA-sensing and RNA-sensing pathways crosstalk? Although KSHV is generally considered to be lymphotropic, numerous cell types are permissive to infection with KSHV; therefore, cell type-specific DNA sensing during infection needs to be fully characterized. Answering these interesting questions would increase our understanding of KSHV–host interactions.

To establish persistent infections and promote viral replication, KSHV has developed several mechanisms to evade the host immune response by interfering with key molecules within the DNA-sensing pathway. The cGAS–STING–TBK1–IRF-interference signaling pathway is the main DNA sensing pathway activated by DNA sensors upon viral infection. KSHV can antagonize these signaling cascades at multiple levels and through various mechanisms. This includes disrupting crucial posttranslational modifications necessary for signaling, such as modifying the phosphorylation or ubiquitination status of signaling molecules. Additionally, viral proteins can also prevent the adequate formation of signaling complexes by steric hindrance. Furthermore, viruses can also promote the degradation of signaling proteins involved in IFN induction. In addition to eliciting the classical type-I IFN signaling pathway, KSHV infection can elicit NF-κB and inflammasome activation, which contribute to restricting viral infection.

Although KSHV utilizes a variety of strategies to antagonize the DNA sensing pathway, there are still unresolved issues that need to be addressed. These include exploring whether KSHV counteracts additional DNA sensors not previously identified in KSHV infections, comprehending the precise mechanisms through which KSHV avoids DNA detection, clarifying whether the strategies employed by KSHV to evade DNA detection remain consistent across various vulnerable cell types, and investigating the potential interaction of these strategies with KSHV-related pathogenesis.

In conclusion, investigating the interplay between DNA-sensing pathways and viral-evasion strategies is crucial for advancing our knowledge of viral infections, immune responses, and host–virus interactions.

## Data Availability

Data are contained within the article.

## References

[1] Takeuchi O., Akira S. (2010). Pattern recognition receptors and inflammation. Cell.

[2] Takaoka A., Wang Z., Choi M.K., Yanai H., Negishi H., Ban T., Lu Y., Miyagishi M., Kodama T., Honda K. (2007). DAI (DLM-1/ZBP1) is a cytosolic DNA sensor and an activator of innate immune response. Nature.

[3] Unterholzner L., Keating S.E., Baran M., Horan K.A., Jensen S.B., Sharma S., Sirois C.M., Jin T., Latz E., Xiao T.S. (2010). IFI16 is an innate immune sensor for intracellular DNA. Nat. Immunol..

[4] Zhang Z., Yuan B., Bao M., Lu N., Kim T., Liu Y.J. (2011). The helicase DDX41 senses intracellular DNA mediated by the adaptor STING in dendritic cells. Nat. Immunol..

[5] Ferguson B.J., Mansur D.S., Peters N.E., Ren H., Smith G.L. (2012). DNA-PK is a DNA sensor for IRF-3-dependent innate immunity. eLife.

[6] Sun L., Wu J., Du F., Chen X., Chen Z.J. (2013). Cyclic GMP-AMP synthase is a cytosolic DNA sensor that activates the type I interferon pathway. Science.

[7] Kim T., Pazhoor S., Bao M., Zhang Z., Hanabuchi S., Facchinetti V., Bover L., Plumas J., Chaperot L., Qin J. (2010). Aspartate-glutamate-alanine-histidine box motif (DEAH)/RNA helicase A helicases sense microbial DNA in human plasmacytoid dendritic cells. Proc. Natl. Acad. Sci. USA.

[8] Latz E., Verma A., Visintin A., Gong M., Sirois C.M., Klein D.C., Monks B.G., McKnight C.J., Lamphier M.S., Duprex W.P. (2007). Ligand-induced conformational changes allosterically activate Toll-like receptor 9. Nat. Immunol..

[9] Soulier J., Grollet L., Oksenhendler E., Cacoub P., Cazals-Hatem D., Babinet P., d’Agay M.F., Clauvel J.P., Raphael M., Degos L. (1995). Kaposi’s sarcoma-associated herpesvirus-like DNA sequences in multicentric Castleman’s disease. Blood.

[10] Chang Y., Cesarman E., Pessin M.S., Lee F., Culpepper J., Knowles D.M., Moore P.S. (1994). Identification of herpesvirus-like DNA sequences in AIDS-associated Kaposi’s sarcoma. Science.

[11] Cesarman E., Chang Y., Moore P.S., Said J.W., Knowles D.M. (1995). Kaposi’s sarcoma-associated herpesvirus-like DNA sequences in AIDS-related body-cavity-based lymphomas. N. Engl. J. Med..

[12] Veettil M.V., Bandyopadhyay C., Dutta D., Chandran B. (2014). Interaction of KSHV with host cell surface receptors and cell entry. Viruses.

[13] Kerur N., Veettil M.V., Sharma-Walia N., Bottero V., Sadagopan S., Otageri P., Chandran B. (2011). IFI16 acts as a nuclear pathogen sensor to induce the inflammasome in response to Kaposi Sarcoma-associated herpesvirus infection. Cell Host Microbe.

[14] Singh V.V., Kerur N., Bottero V., Dutta S., Chakraborty S., Ansari M.A., Paudel N., Chikoti L., Chandran B. (2013). Kaposi’s sarcoma-associated herpesvirus latency in endothelial and B cells activates gamma interferon-inducible protein 16-mediated inflammasomes. J. Virol..

[15] West A.P., Khoury-Hanold W., Staron M., Tal M.C., Pineda C.M., Lang S.M., Bestwick M., Duguay B.A., Raimundo N., MacDuff D.A. (2015). Mitochondrial DNA stress primes the antiviral innate immune response. Nature.

[16] Nishimura K., Ueda K., Sakakibara S., Do E., Ohsaki E., Okuno T., Yamanishi K. (2003). A viral transcriptional activator of Kaposi’s sarcoma-associated herpesvirus (KSHV) induces apoptosis, which is blocked in KSHV-infected cells. Virology.

[17] Härtlova A., Erttmann S.F., Raffi F.A., Schmalz A.M., Resch U., Anugula S., Lienenklaus S., Nilsson L.M., Kröger A., Nilsson J.A. (2015). DNA damage primes the type I interferon system via the cytosolic DNA sensor STING to promote anti-microbial innate immunity. Immunity.

[18] Ishikawa H., Barber G.N. (2008). STING is an endoplasmic reticulum adaptor that facilitates innate immune signalling. Nature.

[19] Saitoh T., Fujita N., Hayashi T., Takahara K., Satoh T., Lee H., Matsunaga K., Kageyama S., Omori H., Noda T. (2009). Atg9a controls dsDNA-driven dynamic translocation of STING and the innate immune response. Proc. Natl. Acad. Sci. USA.

[20] Rathinam V.A., Fitzgerald K.A. (2011). Cytosolic surveillance and antiviral immunity. Curr. Opin. Virol..

[21] Tanaka Y., Chen Z.J. (2012). STING specifies IRF3 phosphorylation by TBK1 in the cytosolic DNA signaling pathway. Sci. Signal..

[22] Ghosh S., May M.J., Kopp E.B. (1998). NF-kappa B and Rel proteins: Evolutionarily conserved mediators of immune responses. Annu. Rev. Immunol..

[23] Napetschnig J., Wu H. (2013). Molecular basis of NF-κB signaling. Annu. Rev. Biophys..

[24] Martinon F., Burns K., Tschopp J. (2002). The inflammasome: A molecular platform triggering activation of inflammatory caspases and processing of proIL-beta. Mol. Cell.

[25] Rathinam V.A., Vanaja S.K., Fitzgerald K.A. (2012). Regulation of inflammasome signaling. Nat. Immunol..

[26] Hemmi H., Takeuchi O., Kawai T., Kaisho T., Sato S., Sanjo H., Matsumoto M., Hoshino K., Wagner H., Takeda K. (2000). A Toll-like receptor recognizes bacterial DNA. Nature.

[27] Kawai T., Sato S., Ishii K.J., Coban C., Hemmi H., Yamamoto M., Terai K., Matsuda M., Inoue J., Uematsu S. (2004). Interferon-alpha induction through Toll-like receptors involves a direct interaction of IRF7 with MyD88 and TRAF6. Nat. Immunol..

[28] Zyzak J., Mitkiewicz M., Leszczyńska E., Reniewicz P., Moynagh P.N., Siednienko J. (2020). HSV-1/TLR9-Mediated IFNβ and TNFα Induction Is Mal-Dependent in Macrophages. J. Innate Immun..

[29] Lund J., Sato A., Akira S., Medzhitov R., Iwasaki A. (2003). Toll-like receptor 9-mediated recognition of Herpes simplex virus-2 by plasmacytoid dendritic cells. J. Exp. Med..

[30] Wang Z., Choi M.K., Ban T., Yanai H., Negishi H., Lu Y., Tamura T., Takaoka A., Nishikura K., Taniguchi T. (2008). Regulation of innate immune responses by DAI (DLM-1/ZBP1) and other DNA-sensing molecules. Proc. Natl. Acad. Sci. USA.

[31] Ishii K.J., Kawagoe T., Koyama S., Matsui K., Kumar H., Kawai T., Uematsu S., Takeuchi O., Takeshita F., Coban C. (2008). TANK-binding kinase-1 delineates innate and adaptive immune responses to DNA vaccines. Nature.

[32] Ablasser A., Bauernfeind F., Hartmann G., Latz E., Fitzgerald K.A., Hornung V. (2009). RIG-I-dependent sensing of poly(dA:dT) through the induction of an RNA polymerase III-transcribed RNA intermediate. Nat. Immunol..

[33] Chiu Y.H., Macmillan J.B., Chen Z.J. (2009). RNA polymerase III detects cytosolic DNA and induces type I interferons through the RIG-I pathway. Cell.

[34] Trapani J.A., Browne K.A., Dawson M.J., Ramsay R.G., Eddy R.L., Show T.B., White P.C., Dupont B. (1992). A novel gene constitutively expressed in human lymphoid cells is inducible with interferon-gamma in myeloid cells. Immunogenetics.

[35] Choubey D., Deka R., Ho S.M. (2008). Interferon-inducible IFI16 protein in human cancers and autoimmune diseases. Front. Biosci. A J. Virtual Libr..

[36] Almine J.F., O’Hare C.A., Dunphy G., Haga I.R., Naik R.J., Atrih A., Connolly D.J., Taylor J., Kelsall I.R. (2017). IFI16 and cGAS cooperate in the activation of STING during DNA sensing in human keratinocytes. Nat. Commun..

[37] Bürckstümmer T., Baumann C., Blüml S., Dixit E., Dürnberger G., Jahn H., Planyavsky M., Bilban M., Colinge J., Bennett K.L. (2009). An orthogonal proteomic-genomic screen identifies AIM2 as a cytoplasmic DNA sensor for the inflammasome. Nat. Immunol..

[38] Fernandes-Alnemri T., Yu J.W., Datta P., Wu J., Alnemri E.S. (2009). AIM2 activates the inflammasome and cell death in response to cytoplasmic DNA. Nature.

[39] Hornung V., Ablasser A., Charrel-Dennis M., Bauernfeind F., Horvath G., Caffrey D.R., Latz E., Fitzgerald K.A. (2009). AIM2 recognizes cytosolic dsDNA and forms a caspase-1-activating inflammasome with ASC. Nature.

[40] Roberts T.L., Idris A., Dunn J.A., Kelly G.M., Burnton C.M., Hodgson S., Hardy L.L., Garceau V., Sweet M.J., Ross I.L. (2009). HIN-200 proteins regulate caspase activation in response to foreign cytoplasmic DNA. Science.

[41] Ishikawa H., Ma Z., Barber G.N. (2009). STING regulates intracellular DNA-mediated, type I interferon-dependent innate immunity. Nature.

[42] Wu J., Sun L., Chen X., Du F., Shi H., Chen C., Chen Z.J. (2013). Cyclic GMP-AMP is an endogenous second messenger in innate immune signaling by cytosolic DNA. Science.

[43] Zhang X., Wu J., Du F., Xu H., Sun L., Chen Z., Brautigam C.A., Zhang X., Chen Z.J. (2014). The cytosolic DNA sensor cGAS forms an oligomeric complex with DNA and undergoes switch-like conformational changes in the activation loop. Cell Rep..

[44] Civril F., Deimling T., de Oliveira Mann C.C., Ablasser A., Moldt M., Witte G., Hornung V., Hopfner K.P. (2013). Structural mechanism of cytosolic DNA sensing by cGAS. Nature.

[45] Li X., Shu C., Yi G., Chaton C.T., Shelton C.L., Diao J., Zuo X., Kao C.C., Herr A.B., Li P. (2013). Cyclic GMP-AMP synthase is activated by double-stranded DNA-induced oligomerization. Immunity.

[46] Kato K., Omura H., Ishitani R., Nureki O. (2017). Cyclic GMP-AMP as an Endogenous Second Messenger in Innate Immune Signaling by Cytosolic DNA. Annu. Rev. Biochem..

[47] Fitzgerald K.A., McWhirter S.M., Faia K.L., Rowe D.C., Latz E., Golenbock D.T., Coyle A.J., Liao S.M., Maniatis T. (2003). IKKepsilon and TBK1 are essential components of the IRF3 signaling pathway. Nat. Immunol..

[48] Sharma S., tenOever B.R., Grandvaux N., Zhou G.P., Lin R., Hiscott J. (2003). Triggering the interferon antiviral response through an IKK-related pathway. Science.

[49] Schmidt A., Rothenfusser S., Hopfner K.P. (2012). Sensing of viral nucleic acids by RIG-I: From translocation to translation. Eur. J. Cell Biol..

[50] Zhang Z., Yuan B., Lu N., Facchinetti V., Liu Y.J. (2011). DHX9 pairs with IPS-1 to sense double-stranded RNA in myeloid dendritic cells. J. Immunol..

[51] Spagnolo L., Rivera-Calzada A., Pearl L.H., Llorca O. (2006). Three-dimensional structure of the human DNA-PKcs/Ku70/Ku80 complex assembled on DNA and its implications for DNA DSB repair. Mol. Cell.

[52] Rivera-Calzada A., Spagnolo L., Pearl L.H., Llorca O. (2007). Structural model of full-length human Ku70-Ku80 heterodimer and its recognition of DNA and DNA-PKcs. EMBO Rep..

[53] Sui H., Zhou M., Imamichi H., Jiao X. (2017). STING is an essential mediator of the Ku70-mediated production of IFN-λ1 in response to exogenous DNA. Sci. Signal..

[54] Burleigh K., Maltbaek J.H. (2020). Human DNA-PK activates a STING-independent DNA sensing pathway. Sci. Immunol..

[55] Ansari M.A., Dutta S., Veettil M.V., Dutta D., Iqbal J., Kumar B., Roy A., Chikoti L., Singh V.V., Chandran B. (2015). Herpesvirus Genome Recognition Induced Acetylation of Nuclear IFI16 Is Essential for Its Cytoplasmic Translocation, Inflammasome and IFN-β Responses. PLoS Pathog..

[56] Bai J., Liu F. (2022). Nuclear cGAS: Sequestration and beyond. Protein Cell.

[57] Gentili M., Lahaye X., Nadalin F., Nader G.P.F., Lombardi E.P., Herve S., De Silva N.S., Rookhuizen D.C., Zueva E., Goudot C. (2019). The N-Terminal Domain of cGAS Determines Preferential Association with Centromeric DNA and Innate Immune Activation in the Nucleus. Cell Rep..

[58] Cui S., Yu Q., Chu L., Cui Y., Ding M., Wang Q., Wang H., Chen Y., Liu X., Wang C. (2020). Nuclear cGAS Functions Non-canonically to Enhance Antiviral Immunity via Recruiting Methyltransferase Prmt5. Cell Rep..

[59] Wang L., Wen M. (2019). Nuclear hnRNPA2B1 initiates and amplifies the innate immune response to DNA viruses. Science.

[60] Lin H., Cao X. (2020). Nuclear innate sensors for nucleic acids in immunity and inflammation. Immunol. Rev..

[61] Jiang Y., Sun S., Quan Y., Wang X., You Y., Zhang X., Zhang Y., Liu Y., Wang B., Xu H. (2023). Nuclear RPSA senses viral nucleic acids to promote the innate inflammatory response. Nat. Commun..

[62] Dutta D., Dutta S., Veettil M.V., Roy A., Ansari M.A., Iqbal J., Chikoti L., Kumar B., Johnson K.E., Chandran B. (2015). BRCA1 Regulates IFI16 Mediated Nuclear Innate Sensing of Herpes Viral DNA and Subsequent Induction of the Innate Inflammasome and Interferon-β Responses. PLoS Pathog..

[63] Iqbal J., Ansari M.A., Kumar B., Dutta D., Roy A., Chikoti L., Pisano G., Dutta S., Vahedi S. (2016). Histone H2B-IFI16 Recognition of Nuclear Herpesviral Genome Induces Cytoplasmic Interferon-β Responses. PLoS Pathog..

[64] Roy A., Ghosh A., Kumar B., Chandran B. (2019). IFI16, a nuclear innate immune DNA sensor, mediates epigenetic silencing of herpesvirus genomes by its association with H3K9 methyltransferases SUV39H1 and GLP. eLife.

[65] Zhang Y., Dittmer D.P. (2018). RIG-I Detects Kaposi’s Sarcoma-Associated Herpesvirus Transcripts in a RNA Polymerase III-Independent Manner. mBio.

[66] Serfecz J.C., Hong Y., Gay L.A. (2022). DExD/H Box Helicases DDX24 and DDX49 Inhibit Reactivation of Kaposi’s Sarcoma Associated Herpesvirus by Interacting with Viral mRNAs. Viruses.

[67] Gay L.A., Sethuraman S., Thomas M., Turner P.C., Renne R. (2018). Modified Cross-Linking, Ligation, and Sequencing of Hybrids (qCLASH) Identifies Kaposi’s Sarcoma-Associated Herpesvirus MicroRNA Targets in Endothelial Cells. J. Virol..

[68] Awasthi S., Verma M., Mahesh A., MI K.K., Govindaraju G., Rajavelu A., Chavali P.L., Chavali S., Dhayalan A. (2018). DDX49 is an RNA helicase that affects translation by regulating mRNA export and the levels of pre-ribosomal RNA. Nucleic Acids Res..

[69] Schumann S., Jackson B.R., Baquero-Perez B., Whitehouse A. (2013). Kaposi’s sarcoma-associated herpesvirus ORF57 protein: Exploiting all stages of viral mRNA processing. Viruses.

[70] Gong D., Kim Y.H., Xiao Y., Du Y., Xie Y., Lee K.K., Feng J., Farhat N., Zhao D., Shu S. (2016). A Herpesvirus Protein Selectively Inhibits Cellular mRNA Nuclear Export. Cell Host Microbe.

[71] Ma Z., Jacobs S.R., West J.A., Stopford C., Zhang Z., Davis Z., Barber G.N., Glaunsinger B.A., Dittmer D.P., Damania B. (2015). Modulation of the cGAS-STING DNA sensing pathway by gammaherpesviruses. Proc. Natl. Acad. Sci. USA.

[72] Kobiler O., Drayman N., Butin-Israeli V., Oppenheim A. (2012). Virus strategies for passing the nuclear envelope barrier. Nucleus.

[73] Wu J.J., Li W., Shao Y., Avey D., Fu B., Gillen J., Hand T., Ma S., Liu X., Miley W. (2015). Inhibition of cGAS DNA Sensing by a Herpesvirus Virion Protein. Cell Host Microbe.

[74] Bhowmik D., Du M., Tian Y., Ma S., Wu J., Chen Z., Yin Q. (2021). Cooperative DNA binding mediated by KicGAS/ORF52 oligomerization allows inhibition of DNA-induced phase separation and activation of cGAS. Nucleic Acids Res..

[75] Yu K., Tian H., Deng H. (2020). PPM1G restricts innate immune signaling mediated by STING and MAVS and is hijacked by KSHV for immune evasion. Sci. Adv..

[76] Weidner-Glunde M., Mariggiò G., Schulz T.F. (2017). Kaposi’s Sarcoma-Associated Herpesvirus Latency-Associated Nuclear Antigen: Replicating and Shielding Viral DNA during Viral Persistence. J. Virol..

[77] Nakajima K.I., Inagaki T., Espera J.M., Izumiya Y. (2024). Kaposi’s sarcoma-associated herpesvirus (KSHV) LANA prevents KSHV episomes from degradation. J. Virol..

[78] Zhang G., Chan B., Samarina N., Abere B., Weidner-Glunde M., Buch A., Pich A., Brinkmann M.M., Schulz T.F. (2016). Cytoplasmic isoforms of Kaposi sarcoma herpesvirus LANA recruit and antagonize the innate immune DNA sensor cGAS. Proc. Natl. Acad. Sci. USA.

[79] Sato M., Suemori H., Hata N., Asagiri M., Ogasawara K., Nakao K., Nakaya T., Katsuki M., Noguchi S., Tanaka N. (2000). Distinct and essential roles of transcription factors IRF-3 and IRF-7 in response to viruses for IFN-alpha/beta gene induction. Immunity.

[80] Sato M., Hata N., Asagiri M., Nakaya T., Taniguchi T., Tanaka N. (1998). Positive feedback regulation of type I IFN genes by the IFN-inducible transcription factor IRF-7. FEBS Lett..

[81] Lin R., Genin P., Mamane Y., Sgarbanti M., Battistini A., Harrington W.J., Barber G.N., Hiscott J. (2001). HHV-8 encoded vIRF-1 represses the interferon antiviral response by blocking IRF-3 recruitment of the CBP/p300 coactivators. Oncogene.

[82] Fuld S., Cunningham C., Klucher K., Davison A.J., Blackbourn D.J. (2006). Inhibition of interferon signaling by the Kaposi’s sarcoma-associated herpesvirus full-length viral interferon regulatory factor 2 protein. J. Virol..

[83] Burysek L., Yeow W.S., Pitha P.M. (1999). Unique properties of a second human herpesvirus 8-encoded interferon regulatory factor (vIRF-2). J. Hum. Virol..

[84] Lubyova B., Pitha P.M. (2000). Characterization of a novel human herpesvirus 8-encoded protein, vIRF-3, that shows homology to viral and cellular interferon regulatory factors. J. Virol..

[85] Hwang S.W., Kim D., Jung J.U., Lee H.R. (2017). KSHV-encoded viral interferon regulatory factor 4 (vIRF4) interacts with IRF7 and inhibits interferon alpha production. Biochem. Biophys. Res. Commun..

[86] Lin S.F., Robinson D.R., Miller G., Kung H.J. (1999). Kaposi’s sarcoma-associated herpesvirus encodes a bZIP protein with homology to BZLF1 of Epstein-Barr virus. J. Virol..

[87] Lefort S., Soucy-Faulkner A., Grandvaux N., Flamand L. (2007). Binding of Kaposi’s sarcoma-associated herpesvirus K-bZIP to interferon-responsive factor 3 elements modulates antiviral gene expression. J. Virol..

[88] Zhu F.X., King S.M., Smith E.J., Levy D.E., Yuan Y. (2002). A Kaposi’s sarcoma-associated herpesviral protein inhibits virus-mediated induction of type I interferon by blocking IRF-7 phosphorylation and nuclear accumulation. Proc. Natl. Acad. Sci. USA.

[89] Liang Q., Fu B., Wu F., Li X., Yuan Y., Zhu F. (2012). ORF45 of Kaposi’s sarcoma-associated herpesvirus inhibits phosphorylation of interferon regulatory factor 7 by IKKε and TBK1 as an alternative substrate. J. Virol..

[90] Yu Y., Wang S.E., Hayward G.S. (2005). The KSHV immediate-early transcription factor RTA encodes ubiquitin E3 ligase activity that targets IRF7 for proteosome-mediated degradation. Immunity.

[91] Zhao Q., Liang D., Sun R., Jia B., Xia T., Xiao H., Lan K. (2015). Kaposi’s sarcoma-associated herpesvirus-encoded replication and transcription activator impairs innate immunity via ubiquitin-mediated degradation of myeloid differentiation factor 88. J. Virol..

[92] Park J., Lee D., Seo T., Chung J., Choe J. (2000). Kaposi’s sarcoma-associated herpesvirus (human herpesvirus-8) open reading frame 36 protein is a serine protein kinase. J. Gen. Virol..

[93] Hwang S., Kim K.S., Flano E., Wu T.T., Tong L.M., Park A.N., Song M.J., Sanchez D.J., O’Connell R.M., Cheng G. (2009). Conserved herpesviral kinase promotes viral persistence by inhibiting the IRF-3-mediated type I interferon response. Cell Host Microbe.

[94] Bisson S.A., Page A.L., Ganem D. (2009). A Kaposi’s sarcoma-associated herpesvirus protein that forms inhibitory complexes with type I interferon receptor subunits, Jak and STAT proteins, and blocks interferon-mediated signal transduction. J. Virol..

[95] Mariggiò G., Koch S. (2017). Kaposi Sarcoma Herpesvirus (KSHV) Latency-Associated Nuclear Antigen (LANA) recruits components of the MRN (Mre11-Rad50-NBS1) repair complex to modulate an innate immune signaling pathway and viral latency. PLoS Pathog..

[96] Choi H.S., Jain V., Krueger B., Marshall V., Kim C.H., Shisler J.L., Whitby D., Renne R. (2015). Kaposi’s Sarcoma-Associated Herpesvirus (KSHV) Induces the Oncogenic miR-17-92 Cluster and Down-Regulates TGF-β Signaling. PLoS Pathog..

[97] Field N., Low W., Daniels M., Howell S., Daviet L., Boshoff C., Collins M. (2003). KSHV vFLIP binds to IKK-gamma to activate IKK. J. Cell Sci..

[98] Ehrlich E.S., Chmura J.C., Smith J.C., Kalu N.N., Hayward G.S. (2014). KSHV RTA abolishes NFκB responsive gene expression during lytic reactivation by targeting vFLIP for degradation via the proteasome. PLoS ONE.

[99] Sakakibara S., Espigol-Frigole G., Gasperini P., Uldrick T.S., Yarchoan R., Tosato G. (2013). A20/TNFAIP3 inhibits NF-κB activation induced by the Kaposi’s sarcoma-associated herpesvirus vFLIP oncoprotein. Oncogene.

[100] Wertz I.E., O’Rourke K.M., Zhou H., Eby M., Aravind L., Seshagiri S., Wu P., Wiesmann C., Baker R., Boone D.L. (2004). De-ubiquitination and ubiquitin ligase domains of A20 downregulate NF-kappaB signalling. Nature.

[101] Seo T., Park J., Lim C., Choe J. (2004). Inhibition of nuclear factor kappaB activity by viral interferon regulatory factor 3 of Kaposi’s sarcoma-associated herpesvirus. Oncogene.

[102] Gregory S.M., Damania B. (2011). Inhibition of the inflammasome response by a viral protein that interacts with NLRs. Commun. Integr. Biol..

[103] Gregory S.M., Davis B.K., West J.A., Taxman D.J., Matsuzawa S., Reed J.C., Ting J.P., Damania B. (2011). Discovery of a viral NLR homolog that inhibits the inflammasome. Science.

[104] Zhang X., Lan Q., Zhang M., Wang F., Shi K., Li X., Kuang E. (2023). Inhibition of AIM2 inflammasome activation by SOX/ORF37 promotes lytic replication of Kaposi’s sarcoma-associated herpesvirus. Proc. Natl. Acad. Sci. USA.

